# The Fate of Threatened Coastal Dune Habitats in Italy under Climate Change Scenarios

**DOI:** 10.1371/journal.pone.0068850

**Published:** 2013-07-09

**Authors:** Irene Prisco, Marta Carboni, Alicia T. R. Acosta

**Affiliations:** Department of Sciences, Roma Tre University, Rome, Italy.; The Ohio State University, United States of America

## Abstract

Coastal dunes worldwide harbor threatened habitats characterized by high diversity in terms of plant communities. In Italy, recent assessments have highlighted the insufficient state of conservation of these habitats as defined by the EU Habitats Directive. The effects of predicted climate change could have dramatic consequences for coastal environments in the near future. An assessment of the efficacy of protection measures under climate change is thus a priority. Here, we have developed environmental envelope models for the most widespread dune habitats in Italy, following two complementary approaches: an “indirect” plant-species-based one and a simple “direct” one. We analyzed how habitats distribution will be altered under the effects of two climate change scenarios and evaluated if the current Italian network of protected areas will be effective in the future after distribution shifts. While modeling dune habitats with the “direct” approach was unsatisfactory, “indirect” models had a good predictive performance, highlighting the importance of using species’ responses to climate change for modeling these habitats. The results showed that habitats closer to the sea may even increase their geographical distribution in the near future. The transition dune habitat is projected to remain stable, although mobile and fixed dune habitats are projected to lose most of their actual geographical distribution, the latter being more sensitive to climate change effects. Gap analysis highlighted that the habitats’ distribution is currently adequately covered by protected areas, achieving the conservation target. However, according to predictions, protection level for mobile and fixed dune habitats is predicted to drop drastically under the climate change scenarios which we examined. Our results provide useful insights for setting management priorities and better addressing conservation efforts to preserve these threatened habitats in future.

## Introduction

Sandy beaches and dunes occur at all latitudes covering ca. 34% of the world’s coastlines [1]. Being a narrow strip between marine and terrestrial ecosystems, coastal dunes are critical habitats characterized by high ecological and biological diversity in terms of their animal and plant communities. Plant communities on sand dunes worldwide show particular adaptations and are spatially arranged along the sea-inland environmental gradient, resulting in a typical vegetation zonation, ranging from the fore dunes near to the sea, to embryo dunes, mobile dunes, transition dunes, fixed dunes and, finally, inland dunes [2,3]. Moreover these habitats provide important ecosystem services to humans, such as coastal protection, erosion control, water catchment and purification, maintenance of wildlife, carbon sequestration, tourism, recreation, education and research [4,5,6]. More than half of the world’s population lives within 60 km of the shoreline [7] and the human pressure on coastal environments has dramatically increased in the last 50 years, especially in the Mediterranean [8]. The rapid increase of a wide range of human activities (urbanization, agriculture, forestry, industry, transport and tourism, etc.) has led to a progressive deterioration and loss of biodiversity, causing fragmentation and a dramatic decline in the distribution and quality of dune habitats [9,10,11]. Coupled with direct anthropogenic impacts on beaches are the effects of predicted climate change, which could have dramatic, widespread and long-lasting consequences for coastal environments in the near future [12,13,14]. Thus, coastal dune management and conservation have become critical issues, representing a priority for many European countries [15,16].

Currently the Habitats Directive (92/43/EEC) is the most important legal instrument for biodiversity conservation at European level [17,18]. It requires each Member State of the European Union to establish a network of special areas for conservation resulting in the Natura 2000 Network sites [19,20]. In addition to a number of species requiring special protection (Annex II), the Directive also lists the habitats of community interest (Annex I), which should be at the center of national conservation efforts and adequately covered by the Natura 2000 Network. Recent assessments have highlighted the critical conservation state of “Coastal sand dunes and inland dunes” habitats included in this list, these being considered among the most endangered, especially in Italy [21]. Notwithstanding recent international conservation efforts, many habitats and species of community interest still fall outside national protected areas. At national level the situation should be viewed with alarm in cases where the protected areas network designed to preserve biodiversity fails to guarantee an effective maintenance of threatened habitats and/or proves to be ineffective in the near future under predicted climate change [9,20,22].

Alterations in climatic and land use conditions can lead to changes in species composition and community structure [23,24]; wildlife conservation faces unprecedented challenges in its response to the accelerated dynamics of global change. In recent years, predictive geographical modeling has increasingly become an important tool for assessing the impact of accelerated climate and other environmental changes (e.g. land use) on the distribution of organisms, so addressing pressing issues in ecology, biogeography, evolution, conservation biology and climate change research [25,26]. Given that, current concepts of nature protection are aimed at habitats in their entirety [20]: habitats as a whole are attracting more attention as focus of research on the effects of global change [27,28,29,30]. Although species-level models have, to date, been the most widely used approach in evaluating changes in biodiversity, other approaches are also available [25,31,32,33]. The choice of modeling (community vs. species) varies depending on the type, quality and quantity of data and on the purpose of the study and the conservation aims [27]. Modeling biodiversity at the community level does not conflict with the species-level approach. Furthermore, it provides a synthesis of the ecological information resulting from a large number of species and restores the biodiversity value in a collective form [27,34]. The community-level approach is therefore particularly valuable for directing national level conservation measures in response to European Union directives. From this perspective, building predictive models for whole plant communities is an important step for the effective management of coastal dune habitats in the near future, particularly in the most endangered portion of their distribution (e.g. in the Mediterranean, and specifically in Italy).

However, several controversial issues arise when attempting to predict future distribution shifts of coastal dune assemblages at a national biogeographical scale. First, coastal dune plant communities are affected simultaneously by a variety of natural and anthropogenic stresses while direct and indirect climate change effects could play a synergical or an antagonistical role in the future scenarios, rendering a comprehensive model over-complicated. As a consequence, any predictive effort is likely to lead to an underestimation of future global change driven range shifts. Second, at a local scale plant species (and community) distribution on coastal dunes is mostly linked to the main sea-inland environmental gradient, this being related to many factors influenced by the distance from the sea such as salt spray, sand burial, wind erosion, substrate stabilization and organic matter content [6,35,36]. But these fine scale variables are often difficult to synthesize and unavailable at coarse biogeographical scales useful for national level planning. In this regard, fine scale dune morphology (e.g. presence of embryo or shifting dunes) is strongly interlinked with the occurrence of coastal dune communities, with dune builder plants in large part determining the geomorphology. Hence, for the sake of model simplicity, all this fine scale complexity can be at least partially conveyed by variables synthesizing sandy shore length, breadth and fragmentation. This is because greater dune surfaces generally provide greater opportunity for mobile and fixed dune formation, while small sandy fragments generally harbor only beach or embryo dune communities.

Another hot debate revolves around the most appropriate species range data to use in modeling. It has been often argued that more accurate results are obtained when the entire climatic niche is taken into account [25]. However, more recently, researchers have highlighted that future conservation needs specific to particular lineages in geographically restricted portions of a species’ range could be better addressed by recognizing intra-specific responses [37]. For example, while several coastal dune species have a pan-European distribution, unique sub-species or phylogeographic lineages occur in the Mediterranean basin, with distinct morphological characteristics, local physiological adaptations and ecological requirements [38,39]. If climate change threatens only local lineages, these threats would go undetected when considering the generic pan-European niche, or unrealistic modeled shifts could be predicted. Hence, national level studies are commonly carried out based on the portion of the range which falls within the national territory [40,41,42]. Regarding all these controversial issues decisions need to be taken and the implications discussed, bearing in mind that any modeling exercise will only produce estimates which make it possible to contrast projected range shifts between communities and set conservation priorities in a relative and comparative way.

In this study we have modeled the response of coastal sandy dune habitats to climate change over the next 50 years, focusing on the case of Italy where coastal dunes require particular attention as highlighted by the latest Italian Report to the European Commission concerning implementation of the Habitats Directive [21]. We adopted a community-level conservation strategy by comparing two complementary modeling approaches: an “indirect” species-based one and a simple “direct” one. In the “indirect” approach we modeled individual plant species distributions as a function of environmental predictors; we then predicted the distribution of the habitat itself based on species occurrences. We compared this “indirect” approach with a straightforward “direct” one, focusing on the habitat as a whole, hereby starting out from unified responses and distribution shifts. The latter could be a realistic approach for coastal dune habitats characterized by a few dominant/diagnostic species. Since it is very difficult to predict how the multiple global change components (e.g. human pressure, sea-level shifts) may in combination affect coastal dune habitats and species, we focused only on direct climate change. Because we were interested in the habitats distribution at national level and we wanted to emphasize the conservation of local lineages and sub-species, we did not consider the whole climatic niche of the dune species but only their restricted distribution.

Finally, we used current and predicted distributions of dune habitats for future global change scenarios in a gap analysis with the Italian network of protected areas in order to evaluate its effectiveness. On such a basis, the specific aims of this study were:

1. To develop distribution models for dune habitats in Italy based on a) diagnostic species’ responses to climate and b) following a direct holistic approach. Is direct modeling of habitat distributions a valid alternative to modeling single species distributions followed by habitat identification?
2. To define the current geographical distribution of dune habitats and analyze how their distribution will be altered under the effects of two climate change scenarios. Which habitats of the coastal zonation will be most at risk?
3. To evaluate the current and future efficacy of the Italian protected areas network. Are dune habitats currently adequately protected? As a consequence of distribution shifts due to climate changes, will the current protected areas network also be effective in future? Which habitats will become less protected?

## Methods

### Study area

This study focused on the entire Italian sandy coastline (about 3’300 Km) [43]. We modeled environmental envelopes for representative coastal dune habitats and species by using a set of selected variables (climate, morpho-sedimentology and land use). In order to facilitate comparisons with other surveys at European level, we transferred and managed all data (habitats, species, environmental variables) and the geographical outputs of the models into a 10 km x 10 km UTM coastal grid, using a GIS software [44]. This resolution is the most commonly used in regional and national analyses of species and habitat distributions [45]. In fact information on the habitat types and species listed in the Annexes of the Habitats Directive [46] has been aggregated at European level and organized on the same UTM grid [47]. We used all the grid cells falling on the coastline in the modeling analysis but, since not every cell along the coast of Italy contains dunes (roughly ¾ do) and the length of the dune systems was variable, we used the length of sandy coast in each grid cell as a correction factor in all models (see below for greater details).

### Habitat and species data

We used information on the current distribution of the most representative dune habitats from an existing database of coastal dune vegetation (VegDunes, EU-IT-005, www.givd.info) [48]. This national database contains phytosociological relevés conducted mostly in the last 30 years throughout Italian Holocenic dune communities, along the whole length of Italian sandy beaches. Each relevé is georeferenced and associated to a habitat type listed in Annex I of the Habitats Directive [46]. The database covers the whole dune zonation, from beach and fore dune habitats to the inner ones. In this study we focused only on those dune habitats of community interest relevant at national level. These coastal habitats are the most widespread along the Italian coasts and have the highest number of records in the database, for a total of 2’483 relevés considered (90% of the VegDunes database). The habitat types selected included beach (Habitat 1210 Annual vegetation of drift lines, 376 relevés), embryo dune (Habitat 2110 Embryonic shifting dunes, 568 relevés), mobile dune (Habitat 2120 Shifting dunes along the shoreline with *Ammophila* *arenaria* , 506 relevés), transition dune (Habitat 2230 *Malcolmietalia* dune grasslands, 333 relevés) and fixed dune (Habitat 2210 *Crucianellionmaritimae* fixed beach dunes, 462 relevés; priority Habitat 2250* Coastal dunes with *Juniperus* spp., 238 relevés). Endemic and/or highly localized habitats, in other words those represented by a low number of relevés, were excluded from the analyses.

**Figure 1:**
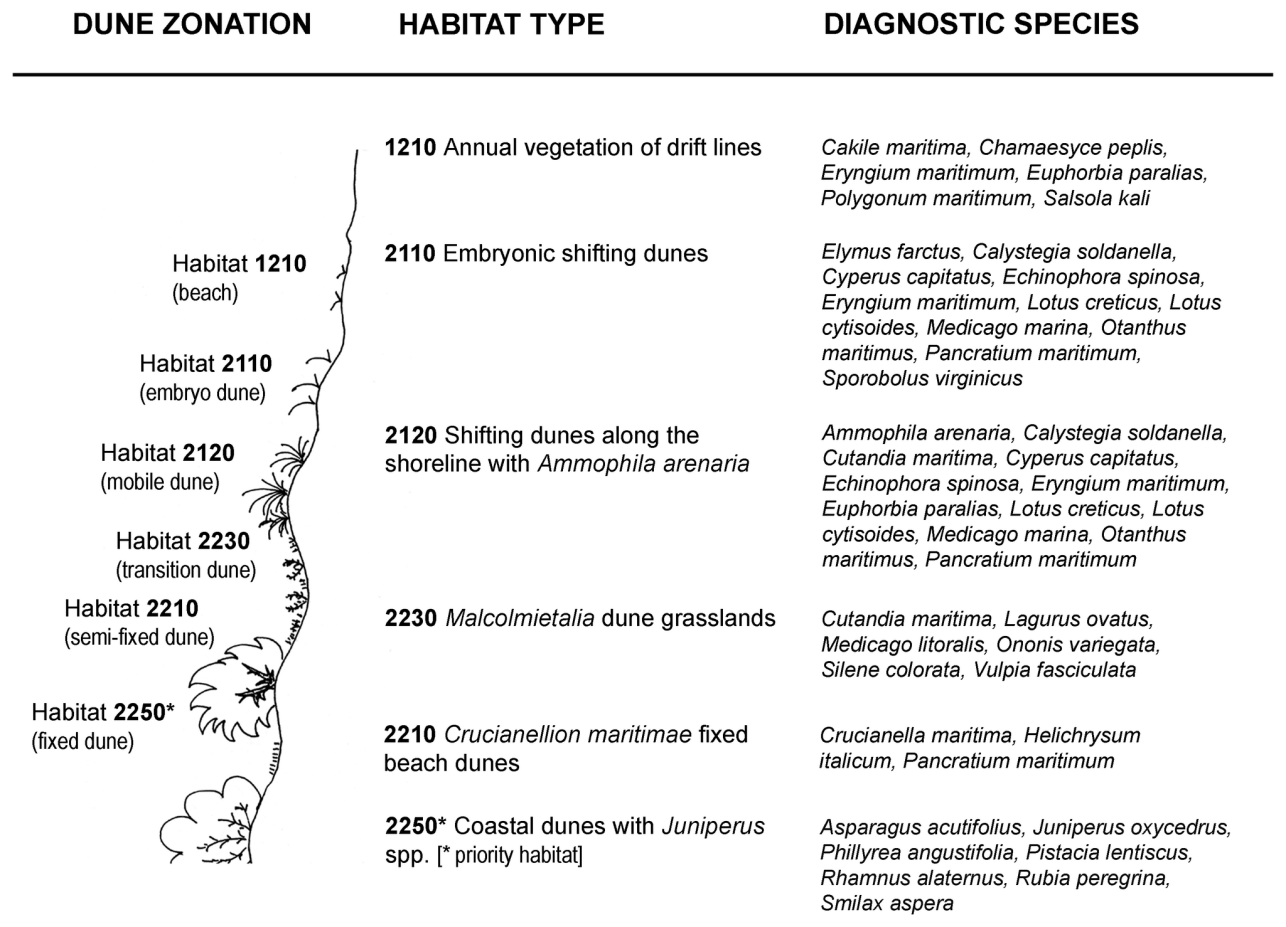
Habitat types and diagnostic species. List of the habitat types considered with corresponding diagnostic plant species (as defined by Interpretation Manuals of Habitats Directive) used in the “indirect models”.

For each habitat type we drew up a list of diagnostic species by using the Italian and the European interpretation manual of the Habitats Directive [49,50]. From the VegDunes database we selected and extracted presence/absence distribution of diagnostic species available with a high number of records (> 40 grid cells) (Figure 1). By relying exclusively on the VegDunes database, even though several coastal species have a wider range (e.g. *Ammophila* *arenaria* or *Cakile* *maritima* , which have a pan-European distribution), we only focus on their Italian distribution. While this is partially a limitation, we consider it appropriate since many pan-European dune species contain distinct Atlantic Ocean/North Sea/Baltic Sea phylogeographic lineages separated from the Mediterranean ones [38,39]. Focusing only on the Italian range avoids biases caused by including the responses of species which only concern the Atlantic or north European sub-species or phylogeographic lineages not occurring in Italy [6,37,50].

### Morpho-sedimentological and land use variables

At local scale dune habitats are affected by several environmental restrictions, such as extreme temperatures, drought, low availability of nutrients, wave and aeolian erosion, salt spray, substrate mobility, etc. [6,35]. At the wider biogeographical scale used in this study we selected two variables that could effectively synthesize the environmental constraints related to the sandy nature of coastal dunes and which are also correlated with fine scale dune morphology: the length of sandy shore and the number of beach fragments. We assume that greater dune surfaces will provide greater opportunity for mobile and fixed dune formation by dune builder plants when these can take root thanks to suitable climatic conditions; thus greater dune surfaces will be able to harbor a higher number of habitats. No data was available on dune breadth, but based on field observations, we assume that longer and less fragmented sandy stretches will tend to be wider as well. In a GIS environment variables were calculated using the Geographical European Coastal Erosion database [51].

The morpho-sedimentological characteristics of each coastal segment in this database are identified by a specific code and nomenclature. We considered 4 “beach” classes: 1) small beaches (200 to 1000 m long) separated by rocky capes (<200m long), 2) extensive beaches (>1 km long) with strands of coarse sediment (gravel or pebbles), 3) extensive beaches (>1 km long) with strands of fine-to-coarse sand and 4) coastlines of soft non-cohesive sediments (barriers, spits, tombolos) [51]. In each grid cell we obtained the “total length of sandy shore” by adding together the length of the 4 beach classes. The variable “number of beach fragments” was introduced in order to quantify the fragmentation degree of sandy beaches due to the presence of rocky, gravel and muddy beaches, estuaries and artificial shoreline (harbors, dikes, quays).

Finally, we considered in the models the urban area in each grid cell as a proxy of human pressure, as this was previously shown to be the most frequent source of degradation, fragmentation and alteration of the current distribution of dune habitats [52]. We calculated the area of “Artificial surfaces” of the CORIN Land Cover 2000 map (first level, code 1) considering a 2 km buffer from the coastline to the inland. Although coastal habitats do not spread for more than 500 meters inland, the presence of human structures (cities, industrial areas, tourist facilities, etc.) up to 2 km can have direct and indirect effects on coastal habitats and diversity [52].

In order to evaluate only the direct effects of climate change on habitats distribution, in our projections for the future we have kept the morpho-sedimentological and land use variables constant.

### Bioclimatic data

We downloaded the 19 WorldClim bioclimatic variables (www.worldclim.org/bioclim) derived from monthly weather station measurements of altitude, temperature, and rainfall [53]. We chose bioclimatic variables because they capture annual ranges, seasonality, and limiting factors useful for niche modeling[53]. . The WorldClim fine resolution (~ 1 km^2^ -30 arc-seconds) data originated from monthly averages of climate as measured at weather stations across the globe, mostly for the 1950–2000 period. These data were afterwards interpolated using the thin-plate smoothing spline algorithm [54,55] creating global climate surfaces for monthly precipitation and minimum, mean and maximum temperature [53].

Since the WorldClim variables are derived from a common set of temperature and precipitation data, they can exhibit multicollinearity [53]. From the full set of variables we removed five precipitation and two temperature variables because they were significantly auto-correlated or they were less meaningful for coastal vegetation communities. For the remaining 12 variables the VIF-approach (Variance Inflation Factor) [56] was used to select the final subset of variables by excluding inter-correlated variables with VIFs > 10 and retaining those deemed more related to coastal vegetation. The final subset of bioclimatic variables was used in all models for all habitats and species, including: the mean diurnal range, the mean temperature of the wettest quarter, the mean temperature of the driest quarter, the mean temperature of the warmest quarter, the precipitation of the wettest quarter and the precipitation of the driest quarter (Tab. 1).

**Table 1:** Variables used in the models.

**Variable name**	**Variable type**	**Reference**
BIO2 Mean diurnal range (Mean of monthly: max temp-min temp)	Bioclimatic	http://www.worldclim.org
BIO8 Mean temperature of wettest quarter	Bioclimatic	http://www.worldclim.org
BIO9 Mean temperature of driest quarter	Bioclimatic	http://www.worldclim.org
BIO10 Mean temperature of warmest quarter	Bioclimatic	http://www.worldclim.org
BIO16 Precipitation of wettest quarter	Bioclimatic	http://www.worldclim.org
BIO17 Precipitation of driest quarter	Bioclimatic	http://www.worldclim.org
Urban area	Land use	CORIN Land Cover 2000
Length of sandy coast	Morpho-sedimentological	Lenôtre et al., 2004
Number of beach fragment	Morpho-sedimentological	Lenôtre et al., 2004

Type and source of the nine variables used in the modeling design. All the variables were calculated in each UTM coastal grid cell.

In order to simulate the distribution of dune habitats under possible future climate conditions, we used the output from the general circulation model HadCM3 (hccpr) from the IPCC 4^th^ Assessment (http://www.ccafs-climate.org/), downscaled with the Delta Method [57]. This downscaling method is based on the interpolation of anomalies (deltas) of the original general circulation model outputs, through the thin-plate smoothing spline algorithm [54,55]. Anomalies are then applied to the WorldClim baseline data [53] at the same spatial resolution (~ 1 km^2^). We focused on predictions for the year 2050 to explore changes in a near and more realistic future. The climate data of this future horizon result from averages across 30 years (2040-2069) [57]. We used two different emission scenarios, A2 and B2. The A2 scenario describes a very heterogeneous world, assuming a high population growth (15 billion by 2100) with an associated increase in emissions and implications for climate change [58]. The B2 scenario describes a world in which the emphasis is on environmental sustainability with a slower population growth, yielding more conservative predictions of anthropogenic emissions [58].

In order to match the resolution of the species and habitats distribution grids we rescaled all the bioclimatic layers (original 1 km^2^ fine resolution) to our UTM coastal grid resolution (10 km x 10 km) by averaging the bioclimatic values within each grid cell in a GIS environment [44].

### Modeling design

We compared two complementary modeling approaches: “indirect” and “direct”. In the “indirect” approach we modeled each habitat in terms of its diagnostic species, following the approach outlined in Bittner et al. [28]. First we modeled the climatic envelope for each species and projected the current and the future occurrence probabilities of the species based on their climatic envelopes. Second, for each habitat, we used the predicted occurrences of its diagnostic species as explanatory variables in the models (instead of climatic variables) to predict habitat distribution both in the current projections and in the future scenarios. In the simple “direct” approach we used the current distribution of the habitat itself and modeled its present and future distribution based on the environmental predictors, as if it possessed an environmental niche as a single entity. This “direct” approach is generally considered highly simplistic, but in the case of coastal dune habitats it might provide a more realistic assumption. In fact, sandy dune communities harbor an extremely specialized flora that includes a small number of species in common with the flora of other terrestrial ecosystems. Furthermore they are often dominated by one or very few characteristic species which do not usually overlap among different habitats [50].

We performed all the projections using eight different and widely used niche-based modeling techniques, within the R-based BIOMOD computational framework [59,60]. This method allows combinations of several modeling techniques in an ensemble forecast resulting in a higher predictive power than when simply taking the average of all models, or using the best model [61,62]. Here we combined the following models: generalized linear models (GLM) [63], generalized boosting models (GBM) [64], generalized additive models (GAM) [65], multivariate adaptive regression splines (MARS) [66], random forest (RF) [67], artificial neural networks (ANN) [68], classification tree analysis (CTA) [69] and surface range envelope (SRE) [70]. Through a randomization procedure BIOMOD makes it possible to estimate the relative importance of each variable by comparing the predictive performance of the models with the observed and with the randomized variable [60]. The predictive performance of the models was evaluated by a random splitting procedure. Specifically we used a random subset (80%) of the distribution data for fitting the models (calibration), and the remainder to evaluate the models (evaluation). To obtain a reliable evaluation of model performance while minimizing the influence of the random splitting of the data, we repeated the calibration/evaluation procedure three times (evaluation runs). We did not select pseudo-absences, as we were fairly confident that absences in the grid cells at this scale represented true absences for these ecosystems (reasonably well studied in Italy). By averaging across evaluation runs and the eight models, the final performance of each model was estimated using TSS (True Skill Statistic). TSS is a statistic that corrects the overall accuracy of model predictions by the accuracy expected to occur by chance: it accounts for commission and omission errors in one parameter and is affected neither by prevalence (proportion of data representing presences) nor by the size of the validation set [71]. Two additional evaluation metrics were calculated for cross-comparison (see Tab. S1). We then applied a binary transformation on the projected occurrence probabilities per grid cell (presence/absence) based on the threshold that maximizes model accuracy using TSS. This threshold represents the optimum correct classification of both presences and absences within the evaluation data [71].

### Gap Analysis

Gap analysis is a method for identifying “gaps” in the network of conservation areas based on the actual spatial distribution of habitats and species, in order to address successful management activities [72,73]. We sought to identify if the considered dune habitats (the most relevant at national level) were sufficiently represented in the Italian conservation areas. Hence we established a “conservation target” defined as the minimum number of grid cells where each habitat occurs, this number of cells having to overlap with protected areas. We established the conservation target following the guide-lines by Rodrigues et al. [73], assuming that each habitat should be represented in the network of protected areas proportionally to its distribution range. A sizeable representation target (a large percentage of the range) should be set for habitats with restricted distribution (e.g. 20%, 30%, 50% or 100% for very rare habitats), while for widespread ones the minimum percentage of conservation target (10% of their distribution) should be sufficient to guarantee the overlapping with protected areas. The issues of how much of habitat’s distribution needs to be represented in protected areas and the arbitrary levels of conservation targets are still under discussion [74,75]. However, we chose approaches for target setting widely used in conservation biology literature [72,73,76,77,78].

We considered all levels of Italian protected areas (National Parks, Regional Parks, Natural Parks, Natural Reserves, Protected Natural Areas, Natural Monuments, Natural Oasis) [79] and the protected Natura 2000 sites (Sites of Community Importance; Habitats Directive [46] - Special Protection Areas; Birds Directive [80]), obtained from the Italian Institute for Environmental Research and Protection (ISPRA, available at: http://www.pcn.minambiente.it/). We calculated the total protected area in each grid cell, considering a 500 meters buffer as the maximum spread of dune habitats towards inland. We established a threshold of 75% of total protected area within this buffer so as to identify each grid cell as protected or not. Considering this threshold, approximately 20% of the total UTM coastal grid would be protected (similar to the total surface currently protected in Italy which is about 20%). For the habitat with the lowest number of presences in the UTM grid (2250* Coastal dunes with *Juniperus* spp.) we chose the arbitrary level of 30% as the conservation target: at least 30% of its distribution range should overlap with the protected areas in order to guarantee the habitat’s conservation. For the most widespread habitat (2230 *Malcolmietalia* dune grasslands) the conservation target was set at 10%. The intermediate values of conservation target for the other habitats were calculated by a simple linear regression.

Finally, the same approach was adopted to evaluate the future efficacy of the protected areas network, based on the predicted distribution of dune habitats in future scenarios.

## Results

In order to provide a quantitative assessment of the climate changes predicted for 2050 along Italian coasts we compared the current climatic conditions within the entire UTM grid with the conditions forecasted for the two future scenarios (Figure S1). All bioclimatic variables were significantly different between the current and the two future scenarios (based on paired Wilcoxon tests). Mean temperatures (BIO 8, 9, 10) are predicted to increase on average between +1.78 °C and +3.85 °C in the A2 scenario, and between +1.59 °C and 3.81 °C in the B2 scenario by 2050. Precipitations are predicted to behave variably with mean increases in the wettest quarter (+11.70 mm in the A2 scenario; +11.23 in the B2 scenario) and mean decreases in the driest quarter (-19.13 mm in the A2 scenario; -15.99 mm in the B2 scenario). For most bioclimatic variables there were only slight differences between the two future scenarios in this short time-range. However, these differences were statistically significant, based on paired Wilcoxon tests, for most variables (but not for precipitations of the wettest quarter). In particular the differences between summer mean temperatures in the two scenarios are predicted to be considerable.

**Figure 2:**
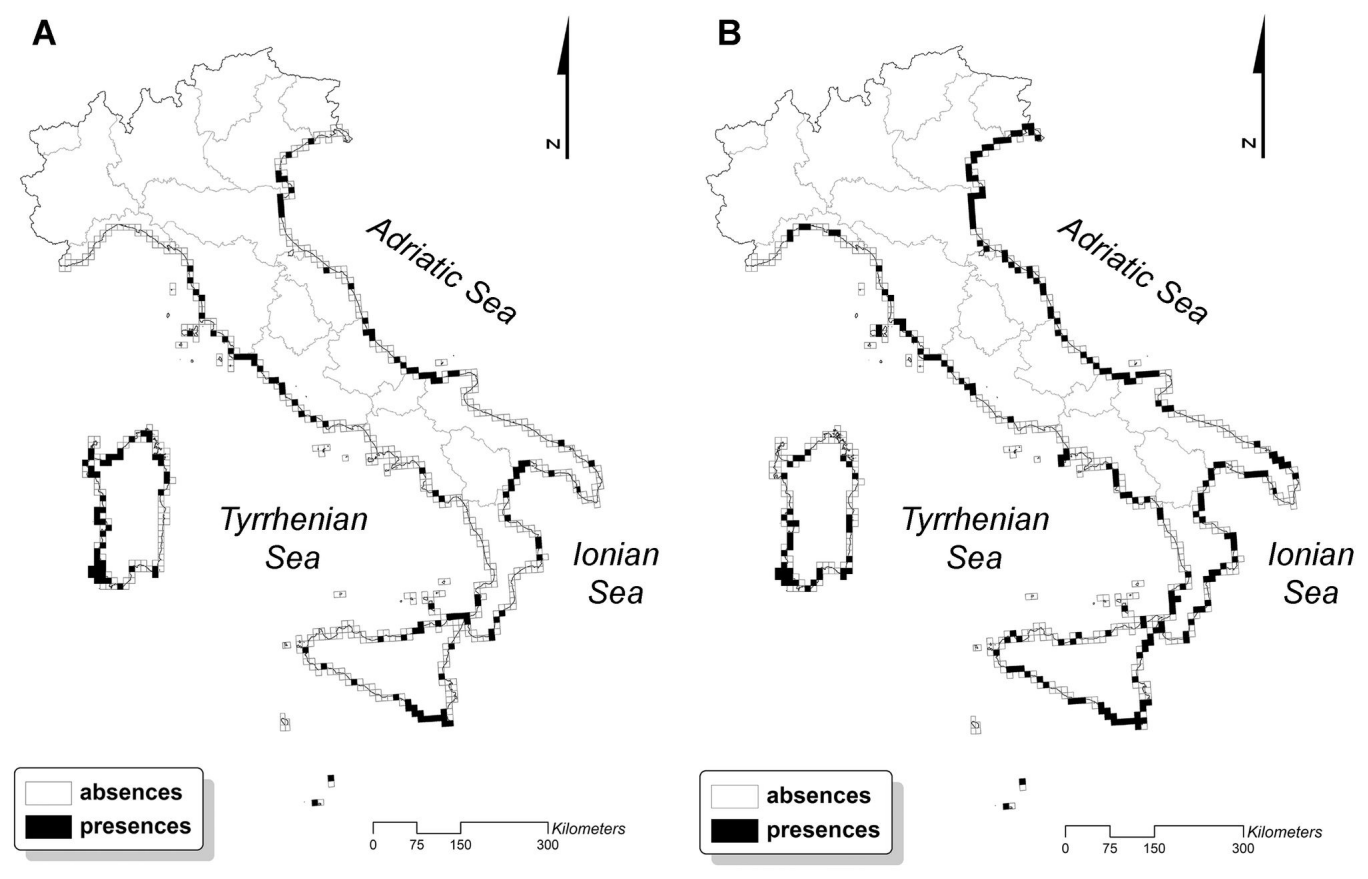
Current and future distribution of embryo dune habitat. (**A**) Current and (**B**) future predicted distribution of one of the habitats close to the coast line: the geographic distribution of embryonic shifting dunes (Habitat 2110) is expected to increase in the near future by more than 40%. As the results of the two future scenarios were similar, only the A2 future scenario is shown (details in Tab. 3).

**Figure 3:**
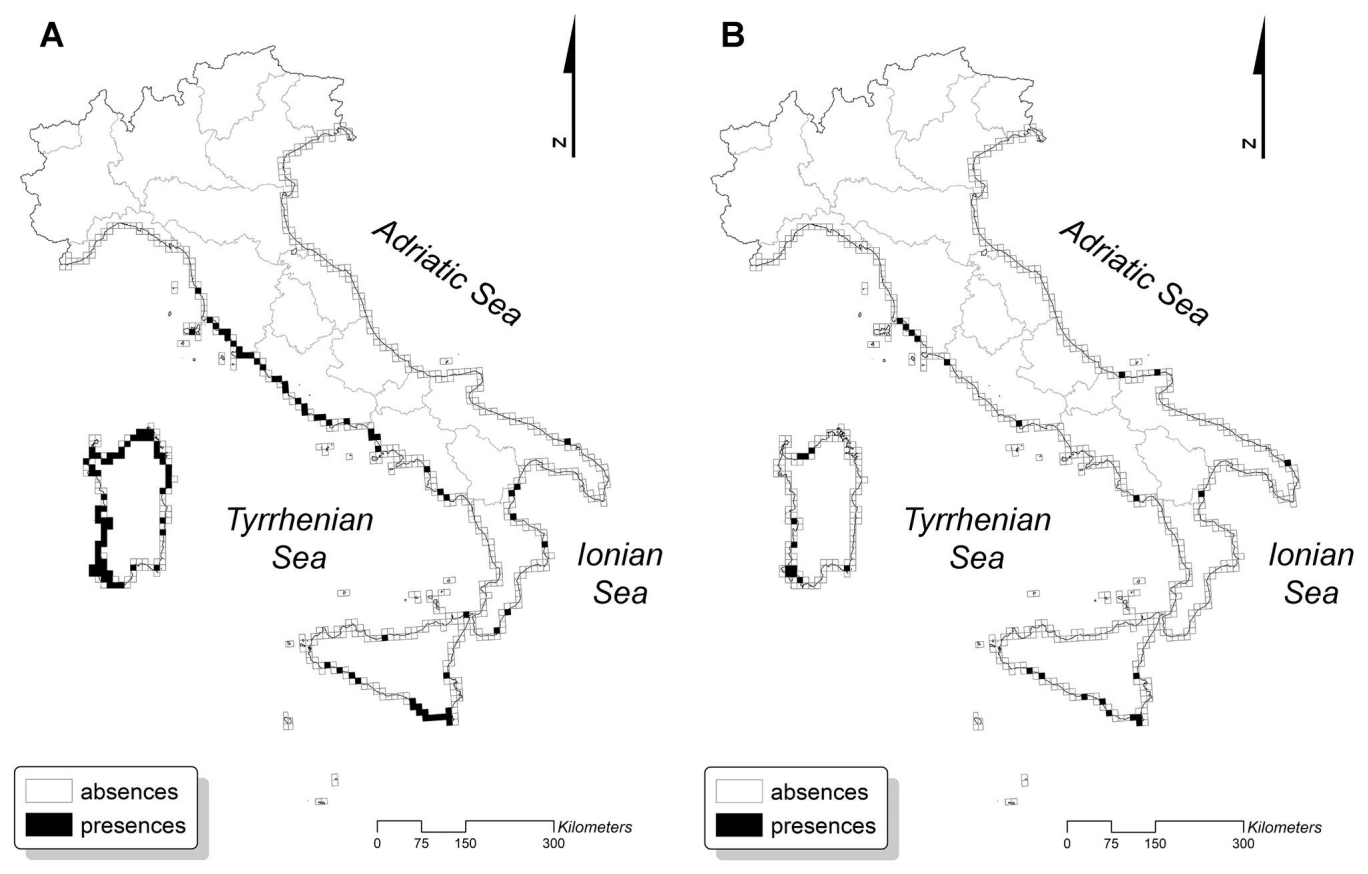
Current and future distribution of fixed dune habitat. (**A**) Current and (**B**) future predicted distribution of one of the fixed dune habitats: the *Crucianellionmaritimae* fixed beach dunes (Habitat 2210) are predicted to lose more than 70% of its current distribution in the near future. As the results of two future scenarios were similar, only the A2 future scenario is shown (details in Tab. 3).

Predictive performance was good in the “indirect” models, with TSS values ranging from 0.68 to 0.81 (Tab. 2 and Tab. S1). By contrast, the evaluation of “direct” models gave TSS values ranging from 0.29 to 0.57, indicating that this is an unreliable approach for coastal dune habitats (see Tab. S2 and Tab. S3 for details). Hence, we reported results only for “indirect” models, discussed here below. There were no significant differences between the two future scenarios (A2 and B2): these showed pretty much the same percentage of increase/decrease of coastal habitats for the year 2050 (Tab. 3). This is likely due to the fact that there were only slight differences in the predicted climatic conditions on Italian coasts for the two scenarios (see Figure S1). The predicted future geographical distribution of coastal dune habitats varied depending on the habitat type. The results showed that the habitats closer to the sea (1210, 2110) may increase their geographical distribution in the near future by even more than 40% (Figure 2). By contrast, mobile dune and fixed dune habitats (2120, 2210, 2250*) are projected to lose most of their current geographical distribution (Figure 3), while the transition dune habitat 2230 is projected to remain stable (Tab. 3). The length of sandy coast was one of the most important variables in all models, followed by the mean temperature of the warmest quarter and the precipitation of the driest quarter (Tab. 2).

**Table 2:** Indirect models evaluation.

**Habitat**	**TSS mean**	**TSS min-max**	**Most important variables**
**1210** Annual vegetation of drift lines	0.68	0.56-0.84	Length of sandy coast; Mean temperature of warmest quarter
**2110** Embryonic shifting dunes	0.72	0.58-0.81	Length of sandy coast; Precipitation of driest quarter
**2120** Shifting dunes along the shoreline with *Ammophila* *arenaria*	0.81	0.62-0.91	Length of sandy coast; Mean temperature of warmest quarter
**2210** *Crucianellionmaritimae* fixed beach dunes	0.81	0.77-0.87	Length of sandy coast; Precipitation of driest quarter
**2230** *Malcolmietalia* dune grasslands	0.68	0.54-0.78	Length of sandy coast
2250* Coastal dunes with *Juniperus* spp. (* priority habitat)	0.80	0.71-0.92	Length of sandy coast; Urban area; Mean diurnal range; Mean temperature of driest quarter

Evaluation by TSS of “indirect models” (species-based) and important variables in the models.

**Table 3:** Indirect models results.

**Habitat**	**Predicted changes Fut. A2**	**Predicted changes Fut. B2**
**1210** Annual vegetation of drift lines	+44.72%	+43.48%
**2110** Embryonic shifting dunes	+48.08%	+40.38%
**2120** Shifting dunes along the shoreline with *Ammophila* *arenaria*	-95.65%	-95.65%
**2210** *Crucianellionmaritimae* fixed beach dunes	-74.59%	-77.05%
**2230** *Malcolmietalia* dune grasslands	+4.49%	+6.18%
2250* Coastal dunes with *Juniperus* spp. (* priority habitat)	-100.00%	-99.03%

**Figure 4:**
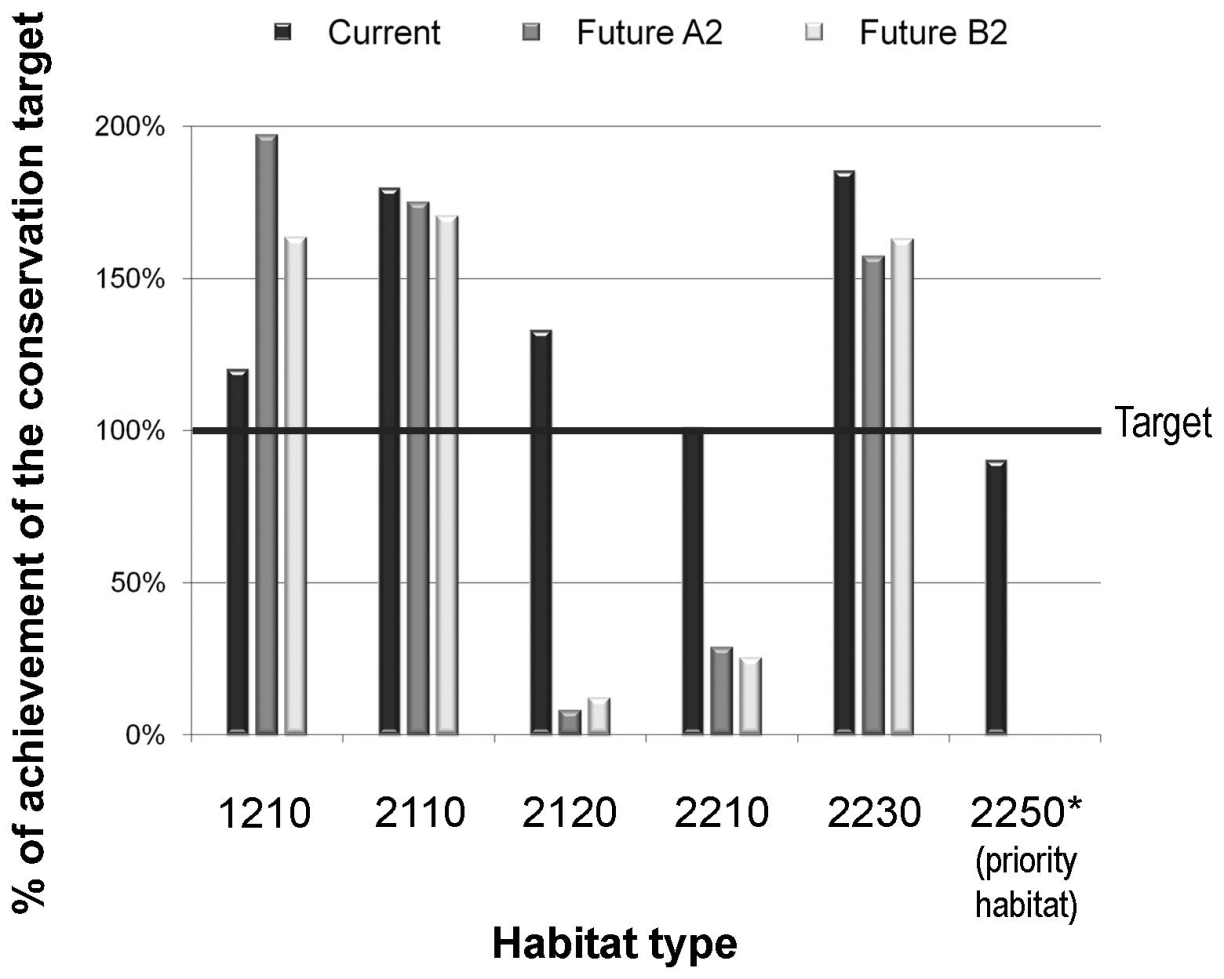
Gap analysis results. Achievement of the conservation target for the selected dune habitats in the current and in the two future climate change scenarios (A2 and B2).

**Figure 5:**
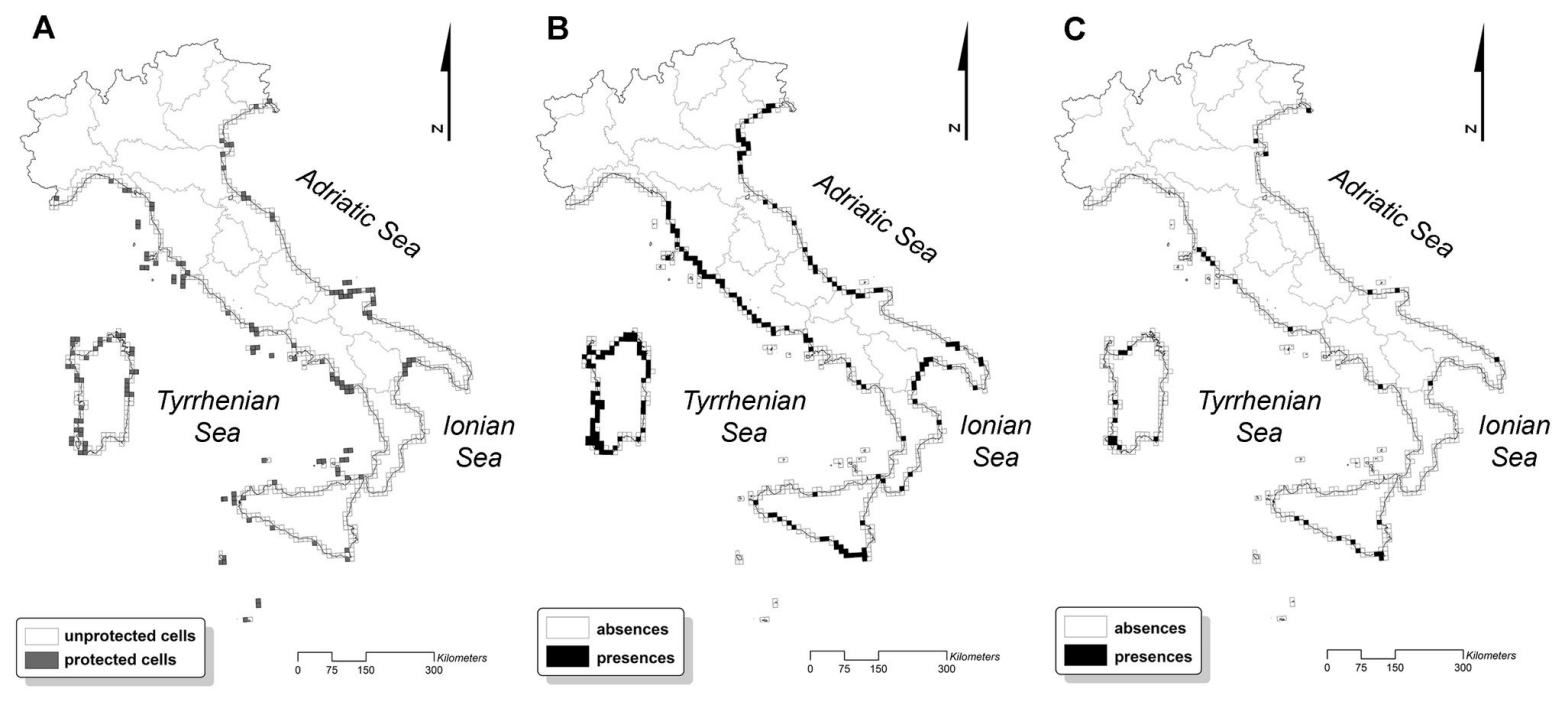
Efficacy of the current protected areas network. Comparison between protected areas network and distribution of the three most threatened dune habitats (2120, 2210, 2250*). (**A**) Protected UTM grid cells; (**B**) UTM grid cells where at least one of the three threatened dune habitats currently occurs; (**C**) UTM grid cells with the predicted future distribution of the three dune habitats (A2 emission scenario).

Gap analysis highlighted that, as of now, the conservation target for almost all dune habitats has been reached in the present. A relevant proportion of current habitats distribution was covered by protected areas, especially for embryo dunes (2110) and transition dunes (2230) which exceeded almost two-fold the target (Figure 4). Beach (1210) and mobile dunes (2120), for their part, achieved the conservation target (120% and 133% respectively), while fixed dunes (2210, 2250*) were at the limit of acceptability (101% and 90% respectively). In the two future scenarios we can observe that beach (1210), embryo dune (2110) and transition dune (2230) habitats will still be adequately protected. More worryingly, the mobile dune (2120) and fixed dune (2210, 2250*) habitats will not reach the conservation target (habitat 2120: 8-12%; habitat 2210: 25-29%; habitat 2250*: 0%) and their level of protection will drop drastically (Figure 4). For mobile and fixed dune habitats, predictions forecasted that there will be a wide range reduction and that the network of protected areas will become ineffective, as shown in Figure 5.

## Discussion

We built habitat distribution models for Italian coastal dune habitats by comparing two approaches, “direct” and “indirect”. We aimed to estimate the response of these threatened habitats to climate change in the near future and to evaluate if dune habitats in Italy are sufficiently represented in the Italian conservation areas, both in the present and in the predicted future distribution.

According to results of the “indirect” models, distribution of the coastal dune habitats closer to the sea will likely increase in the near future. The presence of all coastal dune habitats is strongly dependent on the good quality of sandy littorals, as highlighted by the importance of the length of sandy shore in all models. This is particularly true for beach and embryo dune habitats, such as the annual vegetation of drift lines (Habitat 1210) and the embryonic shifting dunes (Habitat 2110), all being pioneer habitats well-adapted to the strong environmental conditions close to the sea and representing the first stages of the plant colonization [3]. These annual communities were less sensitive to climate change in our projections, probably because they are primarily dependent on the stability of the sea shore. This is not the case for the more inland coastal dune habitats. The *Malcolmietalia* dune grasslands (Habitat 2230) showed no substantial change. This transition habitat is characterized by a mosaic of nitrophilous annual species with ephemeral spring blooming [81], its response to climate change being complex and unpredictable. Mobile dunes and fixed dunes (Habitat 2120, 2210, 2250*), characterized by perennial or semi-perennial plant species and a more complex vegetation structure [81], are projected to lose most of their distribution area in the near future. It is well known that natural stresses, such as wind, sand burial and salt spray, progressively decrease with distance from the sea [82,83,84]. It seems that large scale climatic changes may exert a greater influence on perennial plant species of mobile and fixed dunes than on beach and embryonic dune species, more related to local abiotic factors linked to the closeness of the sea and presence of the sandy substrate [36].

The current network of protected areas is fairly effective in preserving coastal dune habitats; most of them are well represented and meet the conservation target. Only the priority fixed dune habitat 2250* (Coastal dunes with *Juniperus* spp.), in its current distribution, fails to meet such a target, due to its being, at present, less protected than other habitats. It is well-known that over the last 50 years fixed and back dunes have been seriously disturbed by urban development, pine plantation and farming, these all causing coastal juniper woodland regression, especially in the Mediterranean [85,86,87]. However, in the two future scenarios the distribution range reduction of mobile and fixed dune habitats due to climate change could lead to a decrease in the efficacy of existent protected areas. Valuable areas for conservation have already been identified in a previous study [48], pinpointing the high biodiversity value of dune habitats along the northern Adriatic Sea, the two coasts of central Italy and the two major islands, Sicily and Sardinia. A visual scrutiny of the coastal network of protected areas currently reveals some gaps along the Italian shoreline, especially along the Adriatic and Ionian coasts and in southern Sicily. These areas partially overlap with the major concentration of coastal dune habitats and should become priority sites for reserve system implementation. Recently other surveys have evaluated the efficacy of the Natura 2000 Network and protected areas in Italy, e.g. for the conservation of animal biodiversity [78] and from a landscape perspective [88]. These studies have already partially highlighted the need for expanding the current network of coastal conservation areas. Simple local management interventions have also been shown to be effective in limiting human disturbance and promoting vegetation recovery [89]; they can be implemented alongside large scale conservation efforts directed at improving the efficacy of the protected areas network at the national level.

Our results showed that “direct” models had a poor predictive performance. There are intrinsic problems associated with applying habitat models to multi-species assemblages [27]. Habitats are a composition of different plant species that may react differently to changing conditions; their boundaries are not clear cut as there is a gradual transition from one community to another. Shifts or losses of whole habitats are slow events depending on species competition, soil type, dispersal ability, seed production and human impact [28, 90, 91, 92, 93, 94]. Although various authors have obtained valid results by modeling habitats directly [28,29,30,95], in our case modeling dune habitats as single entities has proved to be unsatisfactory. Some coastal dune habitats include a wide number of different phytosociological associations [96]; distinct associations may each respond to climatic variations in their own way. In some cases the communities included under one habitat share only a moderate proportion of common species; this may cause excessive noise in the data, blurring the relationships between environmental and habitat distribution and leading to poor predictive performance of the models. For example the predictive performance of the direct model for the transition dune habitat 2230 (*Malcolmietalia* dune grasslands) is particularly low (see Tab. S2), this habitat being usually described in terms of a wide number of phytosociological associations [96]. Finally, another relevant issue is that all modeling approaches are limited by the quantity and quality of data. Being spatially restricted habitats, coastal dunes cannot be described by a very large amount of data; this makes modeling efforts particularly challenging.

In contrast to the “direct” models, “indirect” models provided a good predictive performance, highlighting the importance of using species’ responses to climate change for modeling these particular habitats. Nevertheless, “indirect” approaches used to predict responses to climate changes of entire habitats have some strengths but also some weaknesses. Models require more time to be implemented; rare species that occur infrequently in the data may not yield accurate results and therefore contribute little to the subsequent community-level approach. Species and other biotic interactions still need to be improved and integrated into the early stages of the models. On the other hand, this community strategy takes into account the individual responses of each species to the environmental predictors and thus is a more realistic representation of community-level shifts.

While our approach is indeed a useful tool for conservation efforts and rapid biodiversity assessment, a few caveats should be borne in mind. Firstly, the species’ responses to climate change could be slightly biased as a consequence of choosing not to consider the full geographic range of the dune species, but only their Italian distribution. As mentioned earlier, this avoids the inclusion of wider environmental tolerances for pan-European species, which, in effect, are limited to the more northern sub-species or phylogeographic lineages. Furthermore we showed that climate on Italian coasts is projected to become hotter and drier in summer: thus it seems unlikely that the Atlantic sub-species will be able to recolonize there or replace the more Mediterranean variants. A greater problem might be posed by the pan-Mediterranean species, in whose case we omitted the southernmost portion of the range. This might have led us to underestimate the ability of these species to tolerate drier and hotter climatic conditions. However the Italian peninsula is to a great degree characterized by a Mediterranean climate, the southern regions and islands being rather extreme. Hence we are reasonably confident that the environmental tolerances of Mediterranean lineages are also well represented.

Secondly, future trends of rising sea level and coastal erosion were not included in our analysis since we aimed to evaluate only direct climate change effects. Coastal environments are dynamic systems responding to variations in sea level, subsidence effects, wave action and sediment transport [97]. Most of the world’s coastlines are in a state of erosion or retreat as a consequence of natural processes, but it remains largely unclear to what extent coastal erosion results from climate change, and to what extent it is associated with relative rises in sea level due to subsidence or human drivers of land loss [98,99]. It is well known that coastal erosion can be caused by local as well as global factors (e.g. rise in sea level due to deglaciation, plate tectonic movements or subsidence effects, etc.) [97]. Although the Mediterranean Sea is among the European low tidal regions with high tectonic activity, the degree of coastal erosion resulting from the rise in sea level is uncertain and the degree of coastal erosion highly variable [100,101]. Thus, attempting to account for such complex and variable developments risked of incorporating too much stochasticity in the models, so leading to less reliable predictions. However we do recognize that beach erosion, new sand delivery and rise in sea level will likely have a major impact, particularly on those species restricted to the most active near beach dune zone. We consider our projections for future distributions of habitats close to the sea to be optimistic, in the sense that their distribution is likely to shrink more than we projected. Whatever the case, our results confirm that at least these habitats will be spared by the effects of direct climate change.

Thirdly, the development of urban areas on coastal dunes was also not taken into account. Human impact on dune habitats depends on the future development of urban planning strategies, even though human development and recreational activities along coasts have been on the increase since 1950. Climate change coupled with urban sprawl will, in all probability, lead to even worse future scenarios. Overall our predicted changes in dune habitats distribution are probably be, if anything, an underestimate. To help prevent such a bleak scenario, recently various efforts have been made to reduce human impact and coastal erosion, e.g. shoreline defense and stabilization [102], development of new laws and institutions for managing coastal land, etc. The inherent complexity of physical processes occurring on coastal dune habitats has limited the accuracy of our predictive models based on climate changes only; nevertheless, given the many uncertainties involved in this regard, our own modeling choices and results could offer an appropriate compromise for directing future conservation efforts.

To conclude, despite some limitations, our results show a clear and reliable future trend for dune habitats linked to shifting climatically suitable areas: as such then, could be useful for environmental management. A worst case scenario is that, without appropriate management, mobile and fixed dune habitats may well disappear as a response to changing climatic conditions alone. Our gap analysis highlights these habitats’ vulnerability not only in the future projections, but also, in some cases, in the immediate present. The results of our habitat distribution models and gap analysis represent useful insights for conservation and management priorities for Italian coastal habitats: indeed, converted into practice, our study could help in drawing up programs to prevent these habitats vanishing completely in future.

## Supporting Information

Figure S1 Climate changes predicted along the Italian coasts for 2050. Comparison between current and future assessment of the bioclimatic variables used in all models on the entire 10 x 10 km grid falling on the coastline. All bioclimatic variables were significantly different between the current and the two future scenarios (paired Wilcoxon tests). The differences between the two future scenarios are slight but also statistically significant for all variables but precipitations of the wettest quarter.
(TIF)

Table S1 Additional evaluation results of the indirect models. Both additional evaluation metrics were calculated using the BIOMOD package. The predictive performance of the “indirect models” (species-based) was good considering each of these methods.
(DOC)

Table S2 Direct models evaluation. Evaluation by TSS of “direct models” (habitat-based) and important variables in the models.
(DOC)

Table S3 Direct models results. Results of the “direct models” (habitat-based) for the year 2050: comparison of percentage changes in dune habitats distribution between the two future scenarios A2 and B2.
(DOC)
