# Supplementary figures and images for: The Fate of Threatened Coastal Dune Habitats in Italy under Climate Change Scenarios
Source: PLoS One. 2013 Jul 9;8(7):e68850. doi: 10.1371/journal.pone.0068850 (PMC3706318; doi:10.1371/journal.pone.0068850)

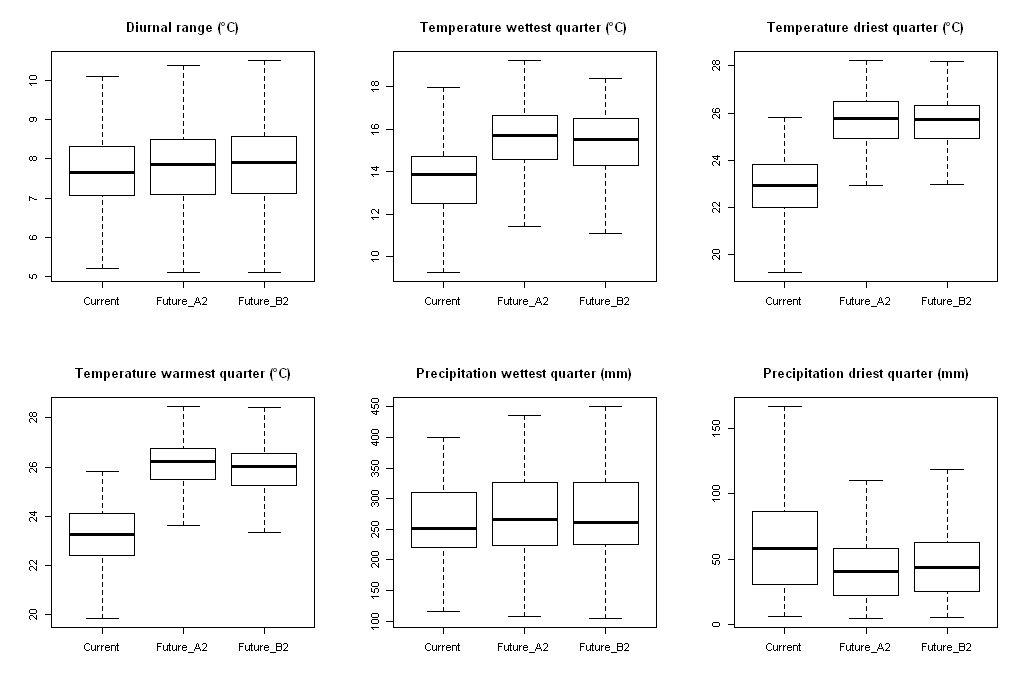

Supplement: Figure S1 — Comparison between current and future assessment of the bioclimatic variables used in all models on the entire 10 x 10 km grid falling on the coastline. All bioclimatic variables were significantly different between the current and the two future scenarios (paired Wilcoxon tests). The differences between the two future scenarios are slight but also statistically significant for all variables but precipitations of the wettest quarter. (TIF) [file pone.0068850.s001.tif]
