# Supplementary material for: The Fate of Threatened Coastal Dune Habitats in Italy under Climate Change Scenarios
Source: PLoS One. 2013 Jul 9;8(7):e68850. doi: 10.1371/journal.pone.0068850 (PMC3706318; doi:10.1371/journal.pone.0068850)
Supplement: Table S1 — Both additional evaluation metrics were calculated using the BIOMOD package. The predictive performance of the “indirect models” (species-based) was good considering each of these methods. (DOC) [file pone.0068850.s002.doc]

**Table S1. Additional evaluation results of the indirect models.**

| **Habitat** | **Kappa mean** | **ROC mean** |
| --- | --- | --- |
| **1210** Annual vegetation of drift lines | 0.60 | 0.89 |
| **2110** Embryonic shifting dunes | 0.61 | 0.89 |
| **2120** Shifting dunes along the shoreline with *Ammophila arenaria* | 0.72 | 0.93 |
| **2210** *Crucianellion maritimae* fixed beach dunes | 0.70 | 0.94 |
| **2230** *Malcolmietalia* dune grasslands | 0.50 | 0.85 |
| **2250*** Coastal dunes with *Juniperus* spp. (* priority habitat) | 0.62 | 0.92 |

Both additional evaluation metrics were calculated using the BIOMOD package. The predictive performance of the “indirect models” (species-based) was good considering each of these methods.
