# Supplementary material for: The Fate of Threatened Coastal Dune Habitats in Italy under Climate Change Scenarios
Source: PLoS One. 2013 Jul 9;8(7):e68850. doi: 10.1371/journal.pone.0068850 (PMC3706318; doi:10.1371/journal.pone.0068850)
Supplement: Table S2 — Evaluation by TSS of “direct models” (habitat-based) and important variables in the models. (DOC) [file pone.0068850.s003.doc]

**Table S2**. Direct models evaluation.

| **Habitat** | **TSS mean** | **TSS min-max** | **Most important variables** |
| --- | --- | --- | --- |
| **1210** Annual vegetation of drift lines | 0.29 | 0.22-0.36 | Length of sandy coast |
| **2110** Embryonic shifting dunes | 0.44 | 0.37-0.50 | Length of sandy coast |
| **2120** Shifting dunes along the shoreline with *Ammophila arenaria* | 0.51 | 0.34-0.62 | Length of sandy coast; Precipitation of driest quarter |
| **2210** *Crucianellion maritimae* fixed beach dunes | 0.57 | 0.37-0.68 | Length of sandy coast; Urban area; Precipitation of driest quarter |
| **2230** *Malcolmietalia* dune grasslands | 0.32 | 0.10-0.49 | Length of sandy coast; Urban area; Precipitation of driest quarter |
| **2250*** Coastal dunes with *Juniperus* spp. (* priority habitat) | 0.49 | 0.28-0.60 | Length of sandy coast; Urban area; Mean temperature of warmest quarter |

Evaluation by TSS of “direct models” (habitat-based) and important variables in the models.
