# Supplementary material for: The Fate of Threatened Coastal Dune Habitats in Italy under Climate Change Scenarios
Source: PLoS One. 2013 Jul 9;8(7):e68850. doi: 10.1371/journal.pone.0068850 (PMC3706318; doi:10.1371/journal.pone.0068850)
Supplement: Table S3 — Results of the “direct models” (habitat-based) for the year 2050: comparison of percentage changes in dune habitats distribution between the two future scenarios A2 and B2. (DOC) [file pone.0068850.s004.doc]

**Table S3**. Direct models results.

| **Habitat** | **Predicted changes Fut. A2** | **Predicted changes Fut. B2** |
| --- | --- | --- |
| **1210** Annual vegetation of drift lines | +0.45% | -0.90% |
| **2110** Embryonic shifting dunes | +15.76% | +15.76% |
| **2120** Shifting dunes along the shoreline with *Ammophila arenaria* | -69.45% | -67.22% |
| **2210** *Crucianellion maritimae* fixed beach dunes | +14.81% | -1.48% |
| **2230** *Malcolmietalia* dune grasslands | -3.89% | -5.00% |
| **2250*** Coastal dunes with *Juniperus* spp. (* priority habitat) | -96.10% | -96.75% |

Results of the “direct models” (habitat-based) for the year 2050: comparison of percentage changes in dune habitats distribution between the two future scenarios A2 and B2.
